# Maximum basal FSH predicts reproductive outcome better than cycle-specific basal FSH levels: waiting for a “better" month conveys limited retrieval benefits

**DOI:** 10.1186/s12958-015-0078-0

**Published:** 2015-08-15

**Authors:** Julian A. Gingold, Joseph A. Lee, Michael C. Whitehouse, Jorge Rodriguez-Purata, Benjamin Sandler, Lawrence Grunfeld, Tanmoy Mukherjee, Alan B. Copperman

**Affiliations:** Reproductive Medicine Associates of New York, 635 Madison Ave 10th Floor, New York, New York 10022 USA; Department of Obstetrics, Gynecology and Reproductive Science, Icahn School of Medicine at Mount Sinai, Klingenstein Pavilion 1176 Fifth Avenue 9th Floor, New York, New York 10029 USA; OB/GYN & Women’s Health Institute, Cleveland Clinic Foundation, 9500 Euclid Ave, Desk A81, Cleveland, OH 44195 USA

**Keywords:** Follicle stimulating hormone, Maximum FSH, Oocyte retrieval outcomes, Cycle cancellation, Cycle delay

## Abstract

**Background:**

Elevated follicle stimulating hormone (FSH) is associated with poor vaginal oocyte retrieval (VOR) outcomes and cycle cancellations but intercycle variability in basal FSH reportedly does not predict ovarian response.

**Methods:**

We conducted a retrospective cohort study of basal FSH (*n* = 15573 cycles) in couples (*n* = 9132) who initiated IVF cycle(s) with basal estradiol (E_2_) <100 pg/mL between 2002 and 2014 to reevaluate this hypothesis.

The most recent (current) FSH, maximum FSH (Max FSH) and prior cycle maximum basal FSH (PMax FSH) were computed for each cycle. Metaphase II (MII) oocyte counts were modeled by age, stimulation type, prior peak E_2_ level, prior MII count, Max FSH, PMax FSH and current FSH. Antral follicle counts, pregnancy, clinical pregnancy and live birth rates were modeled as secondary outcomes.

**Results:**

Max FSH level distinguished completed cycles from cancelled cycles better than PMax FSH or current FSH (AUC of 0.72, 0.71 and 0.61, respectively, *p* < 0.001). Fewer MIIs were retrieved (5.7 ± 3.8) in cycles with Max FSH >13 mIU/mL (*n* = 1475) than those with ≤13 mIU/mL (*n* = 11978) (11.6 ± 7.1) (*p* < 0.001). Max FSH was a better predictor of MII count than PMax FSH or current FSH after controlling for age, stimulation type, prior peak E_2_ level and prior MII count.

Additional MIIs were retrieved on average in cycles with PMax FSH >13 mIU/mL (n = 1930) whose current FSH was ≤13 mIU/ml rather than >13 mIU/ml (*p* < 0.01) after controlling for age, cycle number and stimulation type. However, no improvement in pregnancy or live birth rate was detected.

**Conclusions:**

Max FSH is the best FSH-based predictor of ovarian reserve. Retrieval benefits from waiting for a "better" month appear to exist but are limited.

**Electronic supplementary material:**

The online version of this article (doi:10.1186/s12958-015-0078-0) contains supplementary material, which is available to authorized users.

## Non-technical abstract

Elevated follicle stimulating hormone (FSH) measurements are associated with more frequent in vitro fertilization (IVF) cycle cancellations and fewer oocytes (eggs) being retrieved. It has been thought that a patient with an elevated FSH in the past would not have more oocytes retrieved if her FSH decreased in a subsequent cycle rather than remain elevated. We questioned this belief in a study of over 9132 couples who used IVF between 2002 and 2014. We assessed the effect of a patient's set of FSH measurements on her future outcomes, carefully focusing on 1930 cycles with a previously elevated FSH.

We found that the patient's highest FSH was the best predictor of cycle cancellations and the number of oocytes retrieved. However, even after controlling for the highest FSH, a patient was predicted to obtain slightly more oocytes at retrieval if her FSH decreased. However, we did not detect any improvement in pregnancy or live birth rates.

**Capsule**: Highest historical FSH levels better predict cancellations and VOR counts than cycle-specific FSH, but waiting for a cycle with lower basal FSH still enhances retrieval outcomes.

## Background

Controlled ovarian hyperstimulation (COH) is an essential tool in assisted reproduction, but patient response varies significantly [1, 2]. Ovarian response to gonadotropin stimulation is negatively correlated with basal (early follicular phase) follicle stimulating hormone (FSH) levels [3]. FSH levels correlate with vaginal oocyte retrieval (VOR) outcomes independently of age [4, 5], although their impact on fertilization or implantation rates is at best limited [6–11]. Because of this association, ovarian reserve testing has routinely included basal FSH levels for the past 20 years [12–16].

Precise interpretation of basal FSH measurements has remained elusive. The treatment of patients with diminished ovarian reserve (DOR) remains a persistent challenge [17]. Because collection of additional oocytes (up to ~15) is associated with an increased embryo count and hence live birth rate [18, 19], all avenues for optimizing VOR outcomes, including the timing of ART cycles, are of substantial clinical interest.

Many clinicians have declined to proceed with an IVF cycle following the detection of an elevated basal FSH out of concern for a poor VOR outcome [11, 20, 21]. Increased cycle cancellation rates have been reported in patients with an elevated basal FSH [22].

However, a decision to cancel based on an elevated FSH implicitly assumes that a patient who waits for a "better" month with lower basal FSH levels will have improved VOR outcomes. The existence of a "better" month has never before been rigorously scrutinized, in part because others have claimed that it does not exist [23, 24]. The possible importance of the most recent basal FSH has been called into question by studies demonstrating that a patient's most elevated FSH was at least as good a predictor of ovarian response as the basal FSH in an individual cycle [22–25] and that patients with prior elevations in basal FSH experienced decreased oocyte yield in a subsequent cycle compared to those with normal FSH levels [26].These studies had reported that intercycle variability in basal FSH did not predict ovarian response and could not be used to select an optimal cycle [23]. They also suggested that inclusion of cycle-specific basal FSH conveyed no additional predictive information on ovarian response than using the highest FSH alone [24].

Nonetheless, the high cycle-to-cycle fluctuation of FSH and its association with VOR outcomes leaves open the tantalizing prospect that an ART cycle initiated with a lower basal FSH measurement might be associated with a higher expected VOR outcome. This study sought to challenge the dogma that waiting for a "better" month with improved basal FSH did not enhance outcomes, i.e. no other FSH measurements had predictive value in conjunction with the highest FSH. By representing all of a patient's previous and present FSH elevations as one parameter, the maximum FSH, we could reevaluate whether it predicted VOR better than the current cycle basal FSH. If so, we could directly control for the maximum FSH and model the role of the potentially modifiable current basal FSH on VOR outcomes to give a precise estimate of the benefits associated with waiting for a lower FSH.

## Methods

### Patients

A single-center retrospective cohort study was performed on patients who initiated IVF cycles between January 2002 and March 2014. Study groups were identified from an electronic medical records database according to the patient’s basal FSH history.

### Treatment protocol

IVF stimulation cycles and hormonal adjustments were performed according to standard clinical practice [11]. All cycles were autologous. Patients were treated with one of three different protocols determined by clinician preference: antagonist (ganirelix acetate, Antagon®, Organon USA Inc., Roseland, NJ or cetrorelix acetate, Cetrotide®, EMD Serono, Rockland, MA); downregulation (leuprolide acetate, Lupron®, AbbVie Inc., North Chicago, IL); or microflare (leuprolide acetate, Lupron®, AbbVie Inc., North Chicago, IL). In general, downregulation and antagonist protocols were used in most patients, with the antagonist protocol used specifically in potential hyperresponders and the microflare protocol in poor responders. Total gonadotropin dose was calculated for each patient in IU.

Final oocyte maturation was induced with 6500 IU recombinant hCG alone (Ovidrel®, EMD Serono, Rockland, MA) or, in patients with strong ovarian response or at risk for ovarian hyperstimulation syndrome (OHSS) undergoing an antagonist protocol, with 40 IU of leuprolide acetate together with 1000 IU of hCG (Novarel®, Ferring Pharmaceuticals, Parsippany, NJ) following confirmation of ≥2 mature follicles ≥18 mm by ultrasound. Vaginal oocyte retrieval (VOR) was performed under transvaginal ultrasound guidance 36 h later.

### Clinical management

Basal FSH measurements were routinely taken on Day 3 of the patient's menstrual cycle. For patients who lacked measurements specifically on Day 3, Day 2 or Day 1 measurements were used as substitutes, in that order, for both basal FSH and estradiol (E_2_) (these time points are collectively referred to as "basal" henceforth). FSH measurements with a same-day E_2_ ≥ 100 pg/mL (within 24 h of FSH measurement) were excluded from the study.

Cycle cancellation was recommended when patients had ≤4 mature follicles ≥14 mm and/or >50 % fewer follicles developing than at the same point in a recent (≤1 year prior) cycle; no growing follicles observed during COH in a time frame of 5-6 days; or no increase in E_2_ level between 2 monitoring days at any point during ovarian stimulation.

For each multi-cycle patient at each IVF cycle, the fluctuations in basal FSH were used to calculate 3 parameters: current FSH, maximum FSH (Max FSH) and previous maximum FSH (PMax FSH). "Current FSH" denotes the basal FSH level at the beginning of the given ART cycle. That is, the "current FSH" of a patient with respect to IVF cycle 3 is her basal FSH at the beginning of cycle 3. "Maximum FSH" denotes the maximum of all basal FSH measurements up to and including the given cycle, including cancelled or non-IVF cycles. "Previous maximum" FSH denotes the maximum of all basal FSH measurements prior to the cycle (i.e. excluding any current cycle measurements), including cancelled or non-IVF cycles. Sample calculations of FSH parameters for a set of hypothetical patients are detailed in Table 1. E_2_ levels on the day of ovulatory trigger were recorded as "E_2_ surge". A basal FSH level was considered elevated if it exceeded 13 mIU/mL, according to our standard clinical practice [11].

**Table 1:**
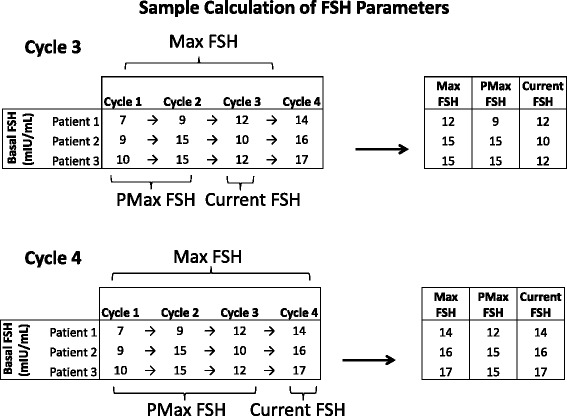
Sample patient FSH parameters and calculation of Max, PMax and current FSH

Consider a set of hypothetical patients whose basal FSH measurements in cycles 1-4 are as shown. Current FSH denotes the basal FSH level at the beginning of the given cycle. Maximum (Max) FSH denotes the maximum of all basal FSH measurements up to and including the given cycle, including cancelled or non-IVF cycles. Previous maximum (PMax) FSH denotes the maximum of all basal FSH measurements prior to the cycle, including cancelled or non-IVF cycles

We will first calculate these values for cycle 3. For patient 1, the current FSH is also the Max FSH (12 mIU/mL). Because the current (most recent) cycle FSH is never considered when calculating PMax, the PMax FSH for patient 1 is the maximum over all previous cycles (9 mIU/mL). For patients 2 and 3, the Max FSH was measured previously and is the same as the PMax FSH. Note that the FSH measurements from cycle 4 play no role in the calculation of FSH parameters for cycle 3

Now consider the same calculations for cycle 4. For patient 1, the new Max FSH is 14 mIU/mL, while PMax FSH is 12 mIU/mL. Note that PMax FSH in the next cycle is the same as Max FSH in the previous cycle. For patients 2 and 3, Max FSH are now from the current cycle (16 and 17 mIU/mL, respectively) while PMax FSH remains 15 mIU/mL

AMH levels were extracted, when available, and were considered to be associated with a given IVF cycle if they were made within the year prior to the ovulatory trigger day of the cycle. For patients with multiple such measurements, the most recent value was used.

Patients were stratified by SART age groups [27] according to the date at which the IVF cycle was initiated: A (≤35 years old (yo)), B (35-38 yo), C (38-41 yo), D (41-43 yo) and E (>43 yo). The time elapsed between the Max FSH measurement and the current FSH was binned as zero months (0-15 d), one month (15-45 d), two months (45-75 d), 2 to 6 months (75-180 d) and more than 6 months (>180 d). For patients whose entire IVF treatment was at our clinic, the IVF cycle number was recorded as 1, 2, 3, 4 or 5+. Number of embryos transferred was recorded was 1, 2, 3, 4 or 5+.

History of prior pregnancy was typically recorded for each patient at intake. Primarily infertility was defined as never having been pregnant at the time of presentation (first IVF treatment). Patients with any prior history of pregnancy at presentation were considered to have secondary infertility.

For patients whose entire infertility treatment was performed at the study's clinic, the time in days between the present IVF cycle and the first cycle (non-IVF or IVF) was calculated. The time in days between the present IVF cycle and the first IVF cycle was also computed.

### Assays

Serum FSH levels were quantitatively assessed by solid-phase, two-site competitive chemiluminescent immunometric assay (Immulite 2000, Siemens, Germany) with an analytical sensitivity of 0.1 mIU/mL and an intra-assay coefficient of variation 2.9–4.2 % for values between 6.8 and 103 mIU/mL. Serum E_2_ levels were quantitatively assessed by solid-phase enzyme-labeled chemiluminescent competitive immunoassay (Immulite 2000, Siemens) with an analytical sensitivity of 15 pg/mL, reportable range up to 2000 pg/mL, and an intra-assay coefficient of variation 4.3–9.9 % for values between 89 and 1800 pg/mL. Serum anti-müllerian hormone (AMH) levels were measured using dual monoclonal antibodies in a chemiluminescent immunoassay (Quest Diagnostics, USA) with an analytical sensitivity of 0.03 ng/mL. Human chorionic gonadotropin (hCG) were quantitatively assessed by solid-phase, two-site competitive chemiluminescent immunometric assay (Immulite 2000, Siemens, Germany) with an analytical sensitivity of 0.4 mIU/mL, reportable range up to 5000 mIU/mL and an intra-assay coefficient of variation 2.5-6.6 % for values between 6.5 and 3,120 mIU/mL. Assays were performed on the same equipment through patient cycles.

### Outcomes

Primary outcome variables were cycle cancellation and metaphase II (MII) oocyte counts. Secondary outcomes were basal antral follicle count (BAFC), pregnancy rates (PRs), clinical PRs and live birth rates (LBRs). A pregnancy was defined as having a serum hCG level exceeding 5 mIU/mL. A clinical pregnancy was defined as the identification of a uterine gestational sac 23-27 days after transfer or 9 days after a positive pregnancy test. LBR was defined as the percentage of all cycles that led to live birth.

Cycle cancellations were predicted based on Max FSH, PMax FSH or current FSH values using a logistic regression model and performance scored with a receiver operating characteristic (ROC) curve. For completed cycles, a Poisson regression generalized estimating equations (GEE) model was created predicting BAFC and MII count by age group, cycle number, stimulation protocol, previous MII count, previous and current cycle E_2_ surge level, previous and current cycle total gonadotropin dosage, AMH levels, Max FSH, PMax FSH, current FSH and time since Max FSH. PR, clinical PR and LBR were modeled with a GEE binomial model by the same parameters as MII count as well as number of embryos transferred, insemination type (conventional insemination vs intracytoplasmic sperm injection (ICSI)) and day of embryo transfer (ET).

### Statistical analyses

Statistical analysis was performed in the R programming language [28]. All two-group comparisons were made by two-sided unpaired T-test. Probability density estimates of FSH parameters were computed with the "density" function in R with default settings. Distributions of cancelled and completed cycle FSH parameters were compared by Kolmogorov-Smirnov statistic. Receiver operating characteristic analysis was performed using the pROC package in R [29]. ROC confidence intervals and comparison between ROC models were calculated using the DeLong method [30]. GEE models were constructed using the R package geepack with an exchangeable correlation structure [31], clustered by patient and ordered by cycle number for each patient. Significant terms from univariate regression models were tested in combination for significance within multivariate models. GEE models were compared using the correlation information criterion (CIC), calculated with the QIC function of the R package MESS [32]. Model significance was tested by chi-square of Wald statistic.

### Ethics, consent and permissions

This research was approved by the Western Institutional Review Board (WIRB). Because of its retrospective, anonymized, aggregated nature, no informed consent was required.

## Results

**Fig. 1:**
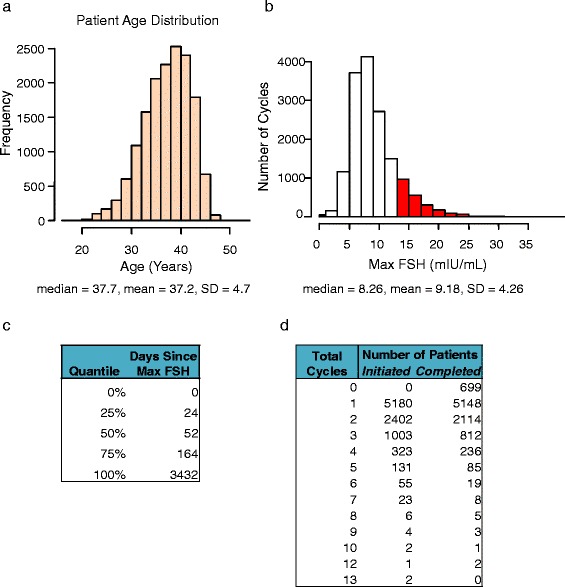
Patient Population Parameters. Histograms of patient **a**) age distribution and **b**) Max FSH distribution. Cycles with Max FSH >13 mIU/mL are shown in red. **c**) Quantiles of time since Max FSH measurement. **d**) Table of number of initiated and completed cycles

A total of 9132 patients initiated 15573 IVF cycles between 2002 and 2014. Patients were 37.2 ±4.7 years old (yo) and had a Max FSH of 9.18 ±4.27 mIU/mL (Fig. 1a-b). Of the completed cycles (*n*=13453), 57.2 % (*n*=7698) were ICSI, 40.5 % (*n*=5448) were conventional insemination, and 2.3 % (*n*=307) were split ICSI and conventional insemination (Table 2, Additional file 1: Table S1). Of the patients with a complete intake history (*n*=6926), 50.6 % (*n*=3504) had a diagnosis of primary infertility, while 49.4 % (*n*=3422) had a diagnosis of secondary infertility (Table 2). The most common causes of infertility across these cycles were male-factor (19.8 %), diminished ovarian reserve (18.2 %), idiopathic (18.2 %) and tubal factor (12.1 %) (Table 2).

**Table 2:** Characteristics of cycles. Cycles were divided by age group, cycle status, cycle number, insemination type, stimulation type, infertility cause, ET day and number of embryos transferred. The number of cycles in each group was noted

	Grouping	Cycles	%
Age Group (yo)	≤35	4835	31.1
	35–38	3312	21.3
	38–41	3745	24.1
	41–43	2197	14.1
	>43	1482	9.5
Cycle Status	Completed	13,453	86.4
	Cancelled	2120	13.6
Cycle Number	1	9132	58.6
	2	3952	25.4
	3	1550	10.0
	4	547	3.5
	5+	392	2.5
Insemination Type	Conventional	5448	40.5
	ICSI	7698	57.2
	Both	307	2.3
Stimulation Type	Antagonist	8023	51.5
	Down Regulation	5005	32.1
	Microflare	2545	16.3
Infertility Cause	Male	3090	19.8
	DOR	2838	18.2
	Idiopathic	2834	18.2
	Tubal	1887	12.1
	Endometriosis	1004	6.4
	PCO	746	4.8
	Ovulatory	718	4.6
	Other	649	4.2
	Not Specified	573	3.7
	Genetic	390	2.5
	Uterine	314	2.0
	RPL	283	1.8
	Endocrine	133	0.9
	Single Woman	114	0.7
Embryo Transfer Day	2	15	0.1
	3	7398	61.2
	4	7	0.1
	5	4407	36.5
	6	261	2.2
Embryos Transferred	1	1542	12.8
	2	4816	39.9
	3	3235	26.8
	4	1590	13.2
	5+	900	7.4

DOR: diminished ovarian reserve

PCO: polycystic ovaries

RPL: recurrent pregnancy loss

For patients (*n*=4025) whose entire infertility treatment (*n*=7195 cycles) was performed at our clinic, IVF cycles were initiated a median of 276 days (50 % within 138-695 days) following the initial non-IVF cycle and a median of the 177 days (50 % within 88-541 days) following the initial IVF cycle.

**Fig. 2:**
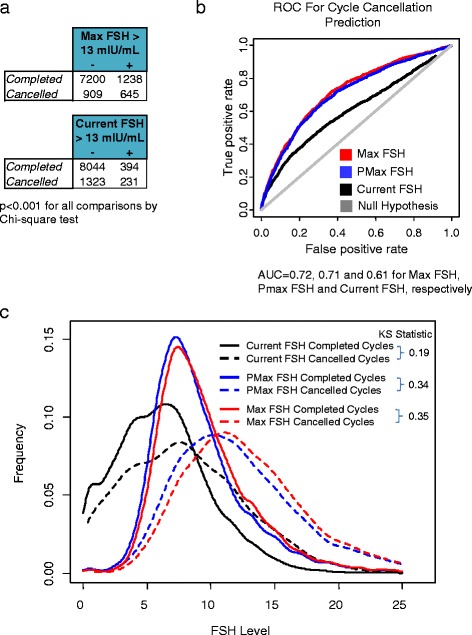
Max FSH predicts cycle cancellations better than current FSH. **a** Number of completed and cancelled cycles with Max FSH and current FSH data, grouped by Max and current FSH elevation status. + indicates true and - indicates false. **b** Receiver operating characteristic (ROC) curve for model prediction of cycle cancellation using Max FSH (red), PMax FSH (blue), current FSH (black) or null hypothesis (grey). **c** Distribution of Max FSH, PMax FSH and current FSH in completed and cancelled cycles. IVF cycles were divided into the subset of completed and cancelled ones and the distribution of their respective Max FSH, PMax FSH and current FSH levels was plotted in red, blue and black, respectively. Completed cycles were plotted in solid lines and cancelled cycles in dashed lines. The Kolmogorov-Smirnov statistic between the distribution parameters for completed and cancelled cycles is noted to the right of each bracketed pair (see Methods for details)

A majority of patients underwent 1 or 2 cycles (57 % (*n*=5180) and 26 % (*n*=2402), respectively). Others underwent more cycles, up to a maximum of 13 (0.02 % (*n*=2)). Fourteen percent of cycles (*n*=2120) were cancelled prior to VOR (Fig. 1d, Table 2). Of these cancelled cycles, 85 % (*n*=1323 of 1554) were in patients with a normal current FSH (≤13 mIU/mL) and 64 % (*n*=1347 of 2120) were in patients with Max FSH ≤13 mIU/mL (i.e. who had never previously experienced an abnormal FSH). Together, 21 % of cancelled cycles were in patients whose FSH was normal at the time of the cycle but abnormal in the past (Fig. 2a).

Of patients with Max FSH >13 mIU/mL at the beginning of their first IVF cycle (*n*=572), 40.7 % (*n*=233) ultimately underwent multiple IVF cycles. Of patients with current FSH >13 mIU/mL at the start of their first IVF cycle (*n*=186), 38.2 % (*n*=71) ultimately underwent multiple IVF cycles. In more than 50 % of cycles, a patient's Max FSH was observed within the previous 2 months (Fig. 1c).

### Max FSH is the best FSH-based predictor of cancellations

The distribution of Max FSH levels differed more between completed and cancelled cycles than did the distribution of either PMax FSH or current FSH level between completed and cancelled cycles (0.35 vs 0.34 or 0.19 Kolmogorov-Smirnov statistic, respectively, *p*<0.001 for all comparisons, larger numbers represent greater differences) (Fig. 2c). ROC analysis was performed to determine the FSH parameters that best predicted cycle cancellations. Max FSH predicted cycle cancellation better than either PMax FSH or current FSH (AUC of 0.72 [0.71–0.74 95 % CI], 0.71 [0.70–0.72]] and 0.61 [0.59–0.62], respectively) (Fig. 2b). The AUC of the Max FSH prediction was significantly better than either the PMax FSH or current FSH prediction (*p*<0.001), and the PMax FSH prediction AUC was significantly better than the current FSH prediction (*p*<0.001). Collectively, Max FSH values better distinguished cancelled from completed cycles than in PMax or current FSH and thus were better predictors of cancellations than either other parameter.

### Max FSH correlates with VOR across age groups better than current FSH

**Fig. 3:**
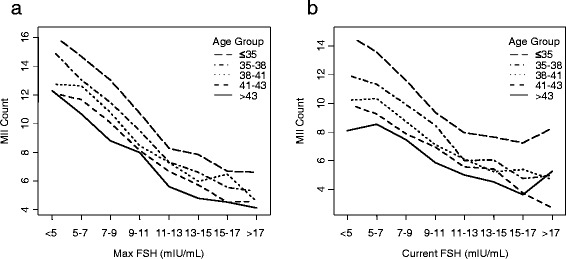
Max FSH and current FSH negatively correlate with MII retrievals. Average number of MII-stage oocytes retrieved, binned both by age group and by either **a**) current FSH level or **b**) Max FSH

Current FSH was negatively correlated with VOR counts when controlled for age group (Fig. 3a). Fewer MIIs (5.5 ±3.5) were retrieved in cycles with current FSH >13 mIU/mL (*n*=394) than those with ≤13 mIU/mL (*n*=8044) (10.4 ±7.1) (*p*<0.001) (Additional file 1: Table S1). Max FSH was also negatively correlated with VOR counts when controlled for age group (Fig. 3b). Fewer MIIs (5.7 ±3.8) were retrieved in cycles with Max FSH >13 mIU/mL (*n*=1475) than those with Max FSH ≤13 mIU/mL (*n*=11978) (11.6 ±7.1) (*p* <0.001) (Additional file 1: Table S1). Fewer MIIs were retrieved in cycles with PMax FSH >13 mIU/mL (*n*=1262) (5.7 ±3.8) than those with ≤13 mIU/mL (*n*=11575) (11.5 ±7.1) (*p*<0.001) (Additional file 1: Table S1).

VOR counts in cycles with Max FSH ≤13 mIU/mL were greater than in cycles with only current FSH ≤13 mIU/mL in all but the <35 yo age group by approximately one MII-stage oocyte (*p*<0.001 for age groups B-E, Additional file 1: Table S1). Thus, cycles in patients over 35 years old yielded more MII oocytes when all previous and current FSH levels were normal than those in which only the current FSH was known to be normal.

### Multiple clinical parameters are individually associated with BAFC and MII

The individual (univariate) association of cycle parameters with BAFC and MII count was explored with a GEE model controlling for patient and cycle number (Table 3). Age group, stimulation protocol, previous MII count, previous and current cycle E_2_ surge level, previous and current cycle total gonadotropin dosage, and AMH levels were associated with both BAFC and MII count (all *p*<0.001) (Table 3). IVF cycle number was associated with MII count but not BAFC (*p*<0.05) (Table 3).

**Table 3:** Individual association of cycle parameters with BAFC and MII count

		BAFC			MII Count		
Parameter	Compared With	Odds Ratio	95 % CI	Sig.	Odds Ratio	95 % CI	Sig.
Age Group B	Age Group A	0.80	[0.75,0.84]	***	0.84	[0.79,0.89]	***
Age Group C	Age Group A	0.68	[0.64,0.72]	***	0.73	[0.69,0.77]	***
Age Group D	Age Group A	0.60	[0.56,0.64]	***	0.64	[0.60,0.69]	***
Age Group E	Age Group A	0.54	[0.51,0.58]	***	0.53	[0.48,0.58]	***
Downregulation Protocol	Antagonist Protocol	1.37	[1.28,1.47]	***	1.16	[1.09,1.23]	***
Microflare Protocol	Antagonist Protocol	0.70	[0.67,0.73]	***	0.72	[0.68,0.76]	***
IVF Cycle 2	IVF Cycle 1	1.00	[0.96,1.04]		0.94	[0.91,0.97]	***
IVF Cycle 3	IVF Cycle 1	0.99	[0.93,1.06]		0.96	[0.91,1.01]	
IVF Cycle 4	IVF Cycle 1	0.97	[0.90,1.05]		0.98	[0.91,1.05]	
IVF Cycle 5+	IVF Cycle 1	0.95	[0.86,1.06]		0.88	[0.79,1.00]	*
Previous MII Count	Per Oocyte	1.04	[1.03,1.04]	***	1.04	[1.04,1.05]	***
E2 Surge Level	Per 1000 pg/mL	1.21	[1.19,1.23]	***	1.40	[1.37,1.42]	***
Previous E2 Surge Level	Per 1000 pg/mL	1.22	[1.18,1.27]	***	1.28	[1.24,1.32]	***
Total Gonadotropin Dosage	Per 1000 IU	0.77	[0.75,0.78]	***	0.82	[0.81,0.83]	***
Previous Total Gonadotropin Dosage	Per 1000 IU	0.80	[0.78,0.83]	***	0.86	[0.84,0.88]	***
AMH	Per 1 ng/mL	1.07	[1.04,1.10]	***	1.09	[1.05,1.12]	***
Max FSH	Per 1 mIU/mL	0.93	[0.93,0.94]	***	0.91	[0.91,0.92]	***
PMax FSH	Per 1 mIU/mL	0.94	[0.93,0.95]	***	0.92	[0.91,0.93]	***
Current FSH	Per 1 mIU/mL	0.98	[0.97,0.98]	***	0.95	[0.94,0.96]	***
Max FSH Observed 1 Month Ago	0 Months Ago	0.98	[0.92,1.04]		1.11	[1.05,1.17]	***
Max FSH Observed 2 Months Ago	0 Months Ago	0.98	[0.91,1.06]		1.06	[0.99,1.14]	
Max FSH Observed 2-6 Months Ago	0 Months Ago	0.97	[0.92,1.02]		1.06	[1.00,1.11]	
Max FSH Observed >6 Months Ago	0 Months Ago	1.01	[0.95,1.07]		1.07	[1.01,1.13]	*

A GEE model controlling for patient and cycle number was constructed to test the association of multiple clinical parameters with BAFC or MII count. Association was noted as odds ratio (OR) with 95 % confidence intervals. Model significance was noted as *** (*p*<0.001) or * (*p*<0.05)

Max FSH, PMax FSH and current FSH were associated with BAFC and MII count. An elevation of 1 mIU/mL in Max FSH was associated with a more decreased MII count than a comparable elevation of PMax FSH or current FSH (odds ratio (OR) 0.91 vs 0.92 and 0.95, *p*<0.05) (Table 3). Time since Max FSH was not associated with BAFC. However, patients whose Max FSH was measured in the previous month or >6 months experienced increased MII counts compared with patients whose Max FSH was made in the same month as their IVF cycle (OR 1.11 and 1.07, respectively) (Table 3).

### Max FSH and current FSH together optimize MII predictions in a combined model

The non-FSH parameters individually associated with MII count were used to construct a combined (multivariate) model of MII count. Age group, cycle number, stimulation type, previous cycle MII count, previous cycle E_2_ surge level and previous cycle total gonadotropin dosage (*n*=1220 cycles in 909 patients) were tested in combination for an association with MII count. Age group, stimulation type, previous cycle MII count, and previous cycle E_2_ surge level remained associated with MII count (CIC score 10.2, *p*<0.001 for each parameter). There was no significant association between cycle number or previous cycle total gonadotropin dose and MII count.

Current FSH, Max FSH and PMax FSH were next added to the combined model. Each parameter remained individually associated with MII count (*p*<0.001). Remaining variability in MII count was better explained by Max FSH than PMax FSH or current FSH (11.9 vs 11.8 or 11.3 CIC score, respectively, larger CIC is better). Inclusion of current FSH into the combined model with Max FSH further improved the model (12.8 CIC score, *p*<0.05). The combined model estimated MII counts as:

$$ \begin{array}{l} \log \left(MII\  count\right)=2.25-\left\{\begin{array}{c}\hfill 0\  if\ \le 35\ yo\hfill \\ {}\hfill 0.02\  if\ \left[35,38\right)\ yo\hfill \\ {}\hfill 0.12\  if\ \left[38,41\right)\ yo\hfill \\ {}\hfill 0.17\  if\ \left[41,43\right)\ yo\hfill \\ {}\hfill 0.25\  if>43\ yo\hfill \end{array}\right.\kern0.75em -\left\{\begin{array}{c}\hfill 0\  if\  Antagonist\hfill \\ {}\hfill 0.02\  if\  Downregulation\hfill \\ {}\hfill 0.13\  if\  Microflare\hfill \end{array}\right.+0.028*MII\  Count\  Prev+0.079\\ {}* Surge\ {E}_2\  Prev\ \left(1000\  pg/ mL\right)-0.029* MaxFSH\ \left(mIU/ mL\right)-0.009\\ {}* CurrentFSH\left(mIU/ mL\right)\end{array} $$

For example, a typical patient (38.3 yo, antagonist cycle, previous cycle MII count of 8, previous cycle E_2_ surge 1560 pg/mL, Max FSH 8.7 mIU/mL, current FSH 6.1 mIU/mL) is predicted to obtain 8.1 MII oocytes in her cycle on average.

### Role of AMH

Because AMH was assessed in relatively few cycles (<11 %), the combined model of MII count did not incorporate AMH levels. However, in a separate multivariate model of MII count in patients with AMH assessments (*n*= 546 cycles in 450 patients), Max FSH significantly improved predictions when combined with age and AMH data compared with using age and AMH alone (*p*<0.001). Use of current FSH values in combination with age, AMH and Max FSH significantly improved the MII prediction compared with age, AMH and Max FSH alone (*p*<0.05), suggesting that AMH measurements are best interpreted in conjunction with both current and Max FSH levels.

### Current FSH improves MII prediction in cycles with elevated PMax FSH

The effect of current FSH was explored in patients with a previously elevated FSH at any point (i.e. PMax FSH >13 mIU/mL). Significantly fewer MII oocytes were retrieved (4.9 ±3.16, *n*=166) in cycles with a current FSH >13 mIU/mL (i.e. FSH was elevated this cycle and in the past) than in cycles with current FSH ≤13 mIU/mL (i.e. FSH was elevated in the past but not this cycle) (5.7 ±3.73, *n*=859) (*p*<0.01) (Fig. 3c), suggesting that current FSH is of continued relevance.

Because there were too few patients with elevated PMax FSH and multiple completed cycles, MII count was modeled using only the cycle-specific parameters of age group, cycle number and stimulation type (*n*=502 cycles in 353 patients). Age group (*p*<0.01), cycle number (*p*<0.05) and stimulation type (*p*<0.001) were significantly associated with MII count in a multivariate model. Max FSH elevation above 13 was not significantly associated with MII count (*p*=0.29) after controlling for age group, cycle number and stimulation type. However, current FSH remained associated with MII count (*p*<0.05). The combined model for cycles with PMax>13 estimated MII counts as:

$$ \begin{array}{l} \log \left(MII\  count\right)=2.13-\left\{\begin{array}{c}\hfill 0\  if\ \le 35\ yo\hfill \\ {}\hfill 0.30\  if\ \left[35,38\right)\ yo\hfill \\ {}\hfill 0.27\  if\ \left[38,41\right)\ yo\hfill \\ {}\hfill 0.40\  if\ \left[41,43\right)\ yo\hfill \\ {}\hfill 0.50\  if>43\ yo\hfill \end{array}\right. + \left\{\begin{array}{c}\hfill 0\  if\  cycle\ 1\hfill \\ {}\hfill 0.12\  if\  cycle\ 2\ \hfill \\ {}\hfill 0.23\  if\  cycle\ 3\hfill \\ {}\hfill 0.33\  if\  cycle\ 4\hfill \\ {}\hfill 0.17\  if\  cycle\ 5+\hfill \end{array}\right.+\left\{\begin{array}{c}\hfill 0\  if\  Antagonist\hfill \\ {}\hfill 0.42\  if\  Downregulation\hfill \\ {}\hfill -0.24\  if\  Microflare\hfill \end{array}\right.-0.015\\ {}* CurrentFSH\left(mIU/ mL\right)\end{array} $$

Applying the combined model, a typical patient with PMax FSH >13 (39.7 yo, antagonist cycle, cycle 1) whose current FSH is 8 mIU/mL is predicted to obtain 5.7 MII oocytes in her cycle on average versus 5.2 MII oocytes if her current FSH were 14 mIU/mL. The resolution of an elevated basal FSH back to normal in a subsequent cycle is predicted to improve VOR counts by approximately 0.5 oocytes on average.

### Multiple clinical parameters are individually associated with pregnancy and live birth rates

The individual (univariate) association of cycle parameters with PR, clinical PR and LBR was explored with a GEE model controlling for patient and cycle number (Table 4). Age group, stimulation protocol, previous MII count, current and previous E_2_ surge level, current and previous total gonadotropin dose, AMH, transfer day and number of embryos transferred were all individually associated with pregnancy and birth rates (Table 4). In contrast, IVF cycle number and insemination type (conventional insemination vs ICSI) were not. Current, Max and PMax FSH were all individually associated with clinical outcomes (Table 4).

**Table 4:** Individual association of cycle parameters with pregnancy rate, clinical pregnancy rate and live birth rate

		Pregnancy rate			Clinical Pregnancy rate			Live Birth rate		
Parameter	Compared with	Odds ratio	95 % CI	Sig.	Odds ratio	95 % CI	Sig.	Odds ratio	95 % CI	Sig.
Age Group B	Age Group A	0.79	[0.65,0.96]	*	0.81	[0.67,0.97]	*	0.81	[0.67,0.99]	*
Age Group C	Age Group A	0.58	[0.48,0.70]	***	0.54	[0.45,0.65]	***	0.46	[0.38,0.56]	***
Age Group D	Age Group A	0.46	[0.37,0.57]	***	0.37	[0.30,0.46]	***	0.31	[0.24,0.39]	***
Age Group E	Age Group A	0.19	[0.15,0.25]	***	0.13	[0.10,0.19]	***	0.09	[0.06,0.14]	***
Downregulation Protocol	Antagonist Protocol	1.65	[1.33,2.06]	***	1.73	[1.41,2.13]	***	1.62	[1.31,2.01]	***
Microflare Protocol	Antagonist Protocol	0.75	[0.64,0.89]	**	0.73	[0.61,0.87]	***	0.73	[0.60,0.89]	**
IVF Cycle 2	IVF Cycle 1	0.87	[0.75,1.00]		0.90	[0.78,1.04]		0.91	[0.78,1.07]	
IVF Cycle 3	IVF Cycle 1	0.91	[0.73,1.13]		0.88	[0.70,1.10]		0.81	[0.63,1.03]	
IVF Cycle 4	IVF Cycle 1	0.81	[0.57,1.13]		0.71	[0.50,1.01]		0.57	[0.39,0.85]	**
IVF Cycle 5+	IVF Cycle 1	0.82	[0.54,1.24]		0.73	[0.48,1.11]		0.68	[0.41,1.13]	
Previous MII Count	Per Oocyte	1.04	[1.02,1.07]	***	1.05	[1.03,1.07]	***	1.03	[1.01,1.05]	**
E2 Surge Level	Per 1000 pg/mL	1.32	[1.23,1.41]	***	1.31	[1.23,1.40]	***	1.24	[1.15,1.33]	***
Previous E2 Surge Level	Per 1000 pg/mL	1.16	[1.03,1.31]	*	1.20	[1.06,1.36]	**	1.11	[0.98,1.27]	
Total Gonadotropin Dosage	Per 1000 IU	0.79	[0.75,0.83]	***	0.76	[0.72,0.80]	***	0.76	[0.71,0.80]	***
Previous Total Gonadotropin Dosage	Per 1000 IU	0.89	[0.82,0.96]	**	0.82	[0.75,0.89]	***	0.81	[0.74,0.89]	***
AMH	Per 1 ng/mL	1.14	[1.04,1.26]	**	1.15	[1.05,1.26]	**	1.08	[0.99,1.17]	
Day 5 Transfer	Day 3 Transfer	1.58	[1.36,1.84]	***	1.70	[1.47,1.97]	***	1.75	[1.50,2.03]	***
Day 6 Transfer	Day 3 Transfer	1.57	[1.04,2.38]	*	1.58	[1.06,2.36]	*	1.67	[1.11,2.50]	*
2 Embryos Transferred	1 Embryo Transferred	2.41	[1.97,2.94]	***	2.49	[2.02,3.06]	***	2.46	[1.97,3.06]	***
3 Embryos Transferred	1 Embryo Transferred	2.60	[2.08,3.24]	***	2.41	[1.92,3.02]	***	2.01	[1.59,2.56]	***
4 Embryos Transferred	1 Embryo Transferred	2.02	[1.55,2.64]	***	2.03	[1.55,2.66]	***	1.97	[1.49,2.61]	***
5+ Embryos Transferred	1 Embryo Transferred	2.56	[1.88,3.49]	***	2.05	[1.50,2.81]	***	1.71	[1.24,2.35]	***
ICSI	Conventional IVF	0.95	[0.83,1.09]		1.00	[0.87,1.15]		1.11	[0.96,1.28]	
Max FSH	Per 1 mIU/mL	0.93	[0.91,0.95]	***	0.93	[0.91,0.95]	***	0.94	[0.92,0.96]	***
PMax FSH	Per 1 mIU/mL	0.93	[0.92,0.95]	***	0.93	[0.91,0.95]	***	0.94	[0.92,0.96]	***
Current FSH	Per 1 mIU/mL	0.94	[0.92,0.96]	***	0.94	[0.92,0.96]	***	0.95	[0.93,0.97]	***
Max FSH Observed 1 Month Ago	0 Months Ago	1.28	[1.06,1.54]	*	1.31	[1.08,1.58]	**	1.18	[0.97,1.45]	
Max FSH Observed 2 Months Ago	0 Months Ago	1.01	[0.79,1.30]		0.93	[0.72,1.20]		0.87	[0.66,1.15]	
Max FSH Observed 2-6 Months Ago	0 Months Ago	0.99	[0.82,1.20]		1.00	[0.83,1.21]		0.93	[0.76,1.14]	
Max FSH Observed >6 Months Ago	0 Months Ago	1.12	[0.93,1.35]		1.20	[0.99,1.44]		1.10	[0.91,1.35]	

A GEE model controlling for patient and cycle number was constructed to test the association of multiple clinical parameters with pregnancy rate, clinical pregnancy rate or live birth rate. Association was noted as odds ratio (OR) with 95 % confidence intervals. Model significance was noted as ***(*p*<0.001), **(*p*<0.01) or *(*p*<0.05)

### FSH is associated with pregnancy rates in a combined model

Clinical outcomes were modeled on the entire cohort of embryo transfers (*n*=3407 cycles) in a combined (multivariate) model. Age group, number of embryos transferred and day of ET were associated with PR (*p*<0.001). Stimulation type and cycle number were not significantly associated with PR. After controlling for these parameters, current FSH but not Max FSH remained weakly associated with PR (OR 0.97 [0.95-0.99], *p*<0.05). Similarly, only age group, number of embryos transferred and day of ET (all *p*<0.001) and current FSH (OR 0.97 [0.95–0.99], *p*<0.05) were associated with clinical PR in a combined model. Age group, number of embryos transferred and day of ET (all *p*<0.001) but not stimulation type or cycle number were associated with the LBR. Neither Max FSH nor current FSH were associated with the LBR after controlling for these parameters.

### Pregnancy and live birth rates not significantly improved in cycles with elevated then improved FSH

To assess the clinical impact of the improved retrieval outcomes observed in cycles with an elevated PMax FSH and improved current FSH, we modeled PR, clinical PR and LBRs using age group, number of embryos transferred, day of ET and Max FSH and current FSH in combination. When restricted to the patients with PMax FSH >13 (*n*=502 transfers), age group, number of embryos transferred and day of ET remained associated with PR, clinical PR and LBRs (*p*<0.01). Neither Max FSH nor current FSH were associated with PR, clinical PR and LBRs after controlling for these parameters.

## Discussion

The study demonstrates that Max FSH predicts both cycle cancellation and MII VOR outcomes better than PMax or current FSH and confirms many existing findings [4, 5, 11, 22–26]. This is the first study demonstrating that current FSH contributes to an assessment of ovarian reserve after controlling for Max FSH. Given the strong association of Max FSH with cancellation rates and VOR outcomes, we advocate the calculation of Max FSH with each cycle and its consideration hand in hand with current FSH. Given the widespread validation of AMH in ovarian reserve testing [33, 34], some clinicians now argue for abandoning FSH altogether in favor of AMH testing [26, 35–37]. However, many others consider current FSH integral for clinical practice, particularly in patients with suspected DOR [38]. The strong connection between FSH and VOR outcomes in this study after controlling for AMH also supports the continued assessment of FSH levels in addition to AMH.

Max FSH has multiple attributes that justify its adoption as the primary FSH-based measure of ovarian reserve. Most significantly, Max FSH is more strongly connected to VOR than PMax or current FSH, confirming previous findings [22–26]. By construction, Max FSH values monotonically increase over time, paralleling the presumed steady decline in ovarian reserve with age. The relative stability of Max FSH versus current FSH addresses one of the key limitations of FSH-based ovarian reserve assessment that has been used to argue against it and in favor of AMH [37]. While the information gleaned from ovarian reserve testing using FSH is complementary to AMH studies, further work is needed to understand how to best combine Max FSH with other ovarian reserve measures and integrate it into routine clinical practice.

Since Max FSH will increase with repeated measurements, patients who undergo additional treatment including FSH measurements will have a higher Max FSH than less frequently measured ones. However, it is unlikely that higher Max FSH levels in patients with poor VOR outcomes can be explained entirely by having undergone more FSH measurements.

The exclusion of all FSH measurements with a same-day E_2_ ≥100 pg/mL was performed in order to ensure a reliable set of basal early follicular phase FSH measurements and to remove the majority of measurements inappropriately labeled as being within in the early follicular phase. These criteria functionally excluded patients undergoing an estrogen priming protocol, during which an otherwise elevated basal FSH would have been falsely masked. Of note, E_2_ levels are consistently <100 pg/mL in healthy patients between D_2_ and D_5_ of their cycles and are commonly elevated at later stages of their menstrual cycle [39]. In addition, cycles with a (non-spurious) basal E_2_ level ≥100 pg/mL are known to have high cancellation rates and poor clinical outcomes [40, 41]. Thus, exclusion of such measurements focuses on the cycles whose outcomes are difficult to predict and not already likely to be cancelled or unsuccessful.

Our ability to estimate the time of infertility was limited by the lack of detailed patient data prior to presentation. While the first IVF cycle was initiated approximately 6 months following an initial non-IVF cycle at our clinic, an infertility patient is not typically evaluated in our practice until she has failed to conceive for at least one year. Thus, total time of infertility before initiating IVF is likely greater than 18 months on average.

As an optimal ovarian hyperstimulation protocol for patients with poor ovarian response has yet to be defined [42], cycle cancellations are often subject to physician preference. Cancellations especially relied on clinician judgment in determining suboptimal ovarian response. The high cancellation rate observed in patients with an elevated FSH and a poor expected ovarian response is unsurprising. However, it is possible that a subset of patients, particularly those with a low Max FSH, may benefit from continuing their IVF cycles until retrieval.

While Max FSH is strikingly more associated with VOR outcomes than current FSH, current cycle FSH is far from irrelevant: the resolution of current FSH from high to normal conveys approximately 0.5 additional MII-stage oocytes of benefit. Thus, we find new evidence to contradict existing dogma that a patient's ovarian response entirely depends on her highest FSH.

Given the particular VOR benefit observed in cycles performed in the month following a Max FSH measurement (Table 3), clinicians seeking to optimize VOR by waiting for a lower basal FSH should be willing to consider waiting at least one month following a new Max FSH measurement. The basal FSH in the cycle following an elevated Max FSH was lower than the previous cycle basal FSH in 173 of 306 cycles (57 %), suggesting that in the short term it is practical to wait for a lower current FSH. Future studies will determine whether particular patient subsets are likely to experience greater VOR benefits by waiting for a “better” month. Circulating antibodies to a wide range of ovarian, adrenocortical, steroidogenic or FSH-related antigens have been associated with DOR [43–45]. Fluctuations in antibody levels may provide one possible explanation for the modestly improved VOR outcomes anticipated in some delayed cycles.

However, the benefits in these patients during a hypothetically more favorable month are limited and must be carefully weighed against their costs. In patients who are able to undergo a limited number of IVF cycles (e.g. due to age, time availability, insurance considerations, etc.), even modestly improved MII oocyte VOR outcomes could justify a delay in stimulation.

Alternatively, in patients with DOR who have a limited number of months of remaining follicular function, lost opportunities for prompt and successful VORs (e.g. in patients in their 40s, with a history of particularly poor outcomes, or an unforeseen requirement for chemotherapy) may justify continued IVF treatment even with the knowledge that VOR counts would be more advantageous in cycles with lower current FSH.

The study failed to demonstrate an improvement in pregnancy, clinical pregnancy or live birth rates for any fluctuations in either Max FSH or current FSH in patients with an elevated PMax FSH. However, Max FSH and current FSH significantly impacted pregnancy and clinical pregnancy rates in the larger cohort. Despite the large original patient cohort, the analysis of particular subsets, e.g. those with AMH measurements, those with elevated PMax FSH, those with multiple IVF cycles performed only at our facility, was frequently impeded by the small patient number. A larger study with improved power may ultimately detect an improvement of LBR in cycles with an improved Max FSH or current FSH after controlling for a multiple clinical parameters. Such a study would be crucial to support the existence of clinical, rather than just embryological, benefits of cycle delay. Alternatively, it is possible that the hypothetical "extra" oocyte retrieved because of improved current FSH was of consistently lower quality than the others that would have been retrieved otherwise and was consistently incapable of sustaining a pregnancy.

An ideal strategy for patients with previously elevated FSH must include consideration of all interventions to improve the total mature oocyte yield. The stimulation protocols for such patients should be tailored to account for their expected poorer VOR outcome. In general, a down regulation protocol with GnRH agonist risks oversuppression of ovarian function, favoring use of a GnRH antagonist protocol, preferably combined with estrogen priming to prevent asynchronous follicle growth [46–51]. However, a meta-analysis associating antagonist protocols with decreased VOR but comparable cancellation and pregnancy rates compared with agonist protocols suggests that agonist protocols might be preferable [52]. Egg banking with multiple stimulation-retrieval cycles can address the poor VOR outcomes per cycle and facilitate retrieval of a desired number of mature oocytes prior to embryo transfer. Preimplantation genetic screening and selective transfer of euploid embryos should be strongly considered in order to avoid riskier multiple pregnancies or delays in reproductive potential from pregnancies by aneuploid embryos. Hence, although further research is necessary to corroborate our findings, a delay in IVF cycle until a "better" month likely conveys a limited improvement in embryologic and possibly clinical reproductive outcome. Decisions to delay cycles are best made in the context of a couple’s detailed fertility history and reproductive goals.

## Conclusions

Max FSH predicts both cycle cancellation and MII VOR outcomes better than PMax or current FSH. In patients with previously elevated basal FSH (PMax >13 mIU/mL), a lower current FSH remains associated with slightly improved MII VOR even after controlling for Max FSH. This improvement is not associated with a detectably improved pregnancy or live birth rate.

## Additional file

Additional file 1: Table S1. Clinical parameters for patients with and without a history of FSH elevation. Cycle data were categorized by presence or absence of elevated current FSH, Max FSH or PMax FSH (>13 mIU/mL) at the time of the cycle and stratified by age group. Incomplete (cancelled) cycles were excluded. Number of cycles with data for each parameter were noted. P-value comparisons were calculated between the elevated and non-elevated groups by t-test. Significance was noted as *** (*p*<0.001), ** (*p*<0.01) or * (*p*<0.05). (XLSX 42 kb)
